# Using transcriptomic and metabolomic data to investigate the molecular mechanisms that determine protein and oil contents during seed development in soybean

**DOI:** 10.3389/fpls.2022.1012394

**Published:** 2022-09-29

**Authors:** Wenjing Xu, Qiong Wang, Wei Zhang, Hongmei Zhang, Xiaoqing Liu, Qingxin Song, Yuelin Zhu, Xiaoyan Cui, Xin Chen, Huatao Chen

**Affiliations:** ^1^ Institute of Industrial Crops, Jiangsu Academy of Agricultural Sciences, Nanjing, China; ^2^ College of Horticulture, Nanjing Agricultural University, Nanjing, China; ^3^ State Key Laboratory of Crop Genetics and Germplasm Enhancement, National Center for Soybean Improvement, Jiangsu Collaborative Innovation Center for Modern Crop Production, Nanjing Agricultural University, Nanjing, China

**Keywords:** protein content, oil content, gene expression pattern, metabolic pathway, molecular regulation, soybean

## Abstract

Soybean [*Glycine max* (L.) Merri.] is one of the most valuable global crops. And vegetable soybean, as a special type of soybean, provides rich nutrition in people’s life. In order to investigate the gene expression networks and molecular regulatory mechanisms that regulate soybean seed oil and protein contents during seed development, we performed transcriptomic and metabolomic analyses of soybean seeds during development in two soybean varieties that differ in protein and oil contents. We identified a total of 41,036 genes and 392 metabolites, of which 12,712 DEGs and 315 DAMs were identified. Analysis of KEGG enrichment demonstrated that DEGs were primarily enriched in phenylpropanoid biosynthesis, glycerolipid metabolism, carbon metabolism, plant hormone signal transduction, linoleic acid metabolism, and the biosynthesis of amino acids and secondary metabolites. K-means analysis divided the DEGs into 12 distinct clusters. We identified candidate gene sets that regulate the biosynthesis of protein and oil in soybean seeds, and present potential regulatory patterns that high seed-protein varieties may be more sensitive to desiccation, show earlier photomorphogenesis and delayed leaf senescence, and thus accumulate higher protein contents than high-oil varieties.

## Introduction

Soybean [*Glycine max* (L.) Merri.] is one of the most valuable global crops. Vegetable soybean is a type of soybean typically harvested during the R6-R7 stage, at which its pods and seeds are suitable for eating (Young et al., 2000). Soybean seeds contain approximately 40% protein and 20% oil (Chaudhary et al., 2015; Li et al., 2015). Optimizing carbon flux towards the synthesis of fatty acids (FAs) and amino acids and improving seed quality has always been a major objective of soybean breeding programs (Bhati et al., 2021). However, the negative correlations between seed protein content with seed oil content and seed yield have hindered progress (Chung et al., 2003; Patil et al., 2017).

Soybean seed oil is mostly comprised of triacylglycerols (TAGs), which have three acyl groups stemming from five fatty acids: linolenic acid, stearic acid, linoleic acid, oleic acid, and palmitic acid (Clemente and Cahoon, 2009). In plants, TAG biosynthesis entails *de novo* FA biosynthesis within plastids as well as TAG assembly in the endoplasmic reticulum (ER) (Bates, 2016; Xu and Shanklin, 2016). The enzyme acetyl-CoA carboxylase initiates the *de novo* FA biosynthesis pathway by converting acetyl-CoA to malonyl-CoA (Salie and Thelen, 2016). The newly synthesized FAs are activated by conversion to FA acyl-CoAs by long-chain acyl-CoA synthetase (LACS), and are then transported to the ER for TAG biosynthesis.

The protein of soybean seeds has 18 amino acids and includes all nine essential amino acids. Of these, there is a deficiency of Cys, Trp, and Met (Zhang et al., 2018). These amino acids are essential for seed development metabolism. While free amino acids (FAAs) are involved in synthesizing seed-storage proteins, one of their most important roles, they are also precursors to the secondary metabolite biosynthesis and provide energy. In addition, amino acids are efficiently catabolized through the tricarboxylic acid (TCA) cycle (Less and Galili, 2009; Kirma et al., 2012).

The levels of individual amino acids vary greatly during seed development. In *Arabidopsis*, young seeds accumulate mostly Ser, Glu, Gln, Gly, and Ala at 6 days after flowering (DAF). However, at 11 DAF, Leu and Val levels significantly increase. Subsequently, higher Ser and Gly levels were observed at 16 DAF (Baud et al., 2002).

Amino acids in plants are synthesized *via* branched pathways (Less and Galili, 2008; Pratelli and Pilot, 2014). The carbon skeleton for Gln, Glu, proline (Pro), and Arg biosynthesis comes from the Krebs cycle intermediate 2-oxoglutarate. The first precursor for synthesizing the following six amino acids is oxaloacetate: methionine (Met), threonine (Thr), asparagine (Asp), isoleucine (Ile), lysine (Lys), and aspartate (Asn). Pyruvate is used to synthesize alanine (Ala), valine (Val), and leucine (Leu), while tryptophan (Trp), tyrosine (Tyr), and phenylalanine (Phe), all of which are aromatic amino acids, are derived from the shikimate pathway. A precursor of serine (Ser) is 3-phosphoglycerate, which leads then to synthesis of glycine (Gly) and cysteine (Cys).

Previous studies used a variety of populations and various mapping methods to identify 248 and 327 quantitative trait loci (QTL) for soybean seed protein and seed oil content, respectively (SoyBase, https://www.soybase.org). Genome-wide association studies (GWAS) were used to find novel loci for soybean oil and protein contents (Li et al., 2018; Lee et al., 2019; Zhang et al., 2021). However, the mechanisms underlying soybean seed development and the regulation of protein and oil biosynthesis have not been comprehensively investigated.

In order to obtain a more complete understanding of the genetic basis of seed oil and protein accumulation in soybean, we conducted transcriptomic and metabolomic analyses of developing soybean seeds. We identified potential key regulators that regulate the biosynthesis of protein and oil in soybean seed and present potential regulatory patterns. Our study provides a valuable resource for the genetic improvement of soybean seed quality through molecular breeding.

## Materials and methods

### Plant materials and tissue preparation

For this experiment, we selected two soybean cultivars, namely, ‘NPS233’ and ‘NPS301’, to evaluate the variations in protein and oil contents. ‘NPS233’ accumulates more protein and less oil in the seeds compared to ‘NPS301’, which is a high oil/low protein variety (**Table 1**). The plant materials were cultivated at the Luhe experimental base of Jiangsu Academy of Agricultural Sciences in Nanjing, China, in the summer of 2021. Seed samples were collected at four developmental stages; 7 DAF (days after flowering), 14 DAF, 21 DAF, and 28 DAF, frozen in liquid nitrogen and stored at -80°C for further metabolite determinations and extraction of RNA.

**Table 1:** Phenotypic differences between ‘NPS233’ and ‘NPS301’.

Material	Leaf type	Whole growth period (day)	HSW (g)	Plant height (cm)	Number of main stem nodes	Oil content (μg/mg)				Protein content (μg/mg)			
						7 DAF	14 DAF	21 DAF	28 DAF	7 DAF	14 DAF	21 DAF	28 DAF
NPS233	oval	105	25.8	69	18	69.6	73.0	65.9	81.9	42.3	116.7	151.7	205.6
NPS301	circular	120	20.1	44	12	75.6	94.3	126.5	124.1	33.9	73.5	120.0	169.5

HSW, Hundred-seed weight; DAF, days after flowering.

### Measurement of protein and oil

For measurement of samples protein and oil content (%), ~20 g seeds were grounded to powder and analyzed by Kjeldahl Method and Soxhlet extraction, respectively (Bremner, 1960; Fehr et al., 1968). Samples protein and oil content (%) were averaged over three replications.

### Extracting of metabolites

Biological samples were vacuum freeze-dried with a lyophilizer (Scientz-100F) and then crushed with a mixer mill (MM 400, Retsch) and a zirconia bead at 30 Hz for 1.5 min. 100 mg samples of lyophilized powder were dissolved in a solution of 1.2 ml 70% methanol, vortexed every 30 minutes for 30 seconds (six total vortexes), and maintained overnight at 4°C. Extracts were filtered (SCAA-104, 0.22μm pore size; ANPEL, Shanghai, China; http://www.anpel.com.cn/) after they were centrifuged for 10 min at 16,000 g. They were then used in UPLC-MS/MS analysis.

### UPLC conditions

Samples were analyzed with a UPLC-ESI-MS/MS system (UPLC, Shimadzu Nexera X2, www.shimadzu.com.cn/; MS, Applied Biosystems 4500 Q TRAP, www.appliedbiosystems.com.cn/) using the following conditions: UPLC; the column was an Agilent SB-C18 (1.8 µm, 2.1 mm×100 mm); mobile phase used solvent A, pure water with 0.1% formic acid, and solvent B, and acetonitrile with 0.1% formic acid. Samples were separated using a gradient program starting at 95% A, and 5% B. After 9 min, a linear gradient to 5% A, 95% B was set, and a composition of 5% A, 95% B was maintained for 1 min, after which a composition of 95% A, 5% B was attained after 1.10 min and maintained for 2.9 min. The flow velocity, column oven temperature, and injection volume were 0.35 ml per minute, 40°C, and 4 μl, respectively. An ESI-triple quadrupole-linear ion trap (QTRAP)-MS was connected with the effluent.

### ESI-Q TRAP-MS/MS

Triple quadrupole (QQQ) and LIT were obtained using a triple quadrupole-linear ion trap mass spectrometer (Q TRAP), AB4500 Q TRAP UPLC/MS/MS System, with an ESI Turbo Ion-Spray interface that was placed in positive and negative ion modes and operated using Analyst 1.6.3 software (AB Sciex). The parameters of the ESI source operation included the following: ion source, turbo spray; source temperature, 550°C; ion spray voltage, (IS) 5500 V (positive ion mode)/-4500 V (negative ion mode); ion source gas I (GSI), gas II (GSII), and curtain gas (CUR) were set at 50, 60, and 25.0 psi, respectively; collision-activated dissociation (CAD) was high. Instrument tuning and mass calibration were carried out using 10 and 100 μmol/L polypropylene glycol solutions in QQQ and LIT modes, respectively. QQQ scans were obtained with MRM experiments with collision gas (nitrogen) set to medium. DP and CE for individual MRM transitions were performed using additional optimization of DP and CE. Certain MRM transitions were observed during each time period according to the eluted metabolites within that time frame.

### Principal component analysis

PCA was not supervised and was carried out with the statistics function prcomp in R (www.r-project.org). Prior to this analysis, the data were subjected to unit variance scaling.

### Pearson correlation coefficients and hierarchical cluster analysis

Sample and metabolite HCA results are displayed as heatmaps with dendrograms, and the Pearson correlation coefficients (PCC) of the samples were assessed using the cor function in R and only displayed as heatmaps. The R package pheatmap was used to perform both PCC and HCA. For the HCA, a color spectrum was used to display the normalized metabolite signal intensities with unit variance scaling.

### Identifying differential metabolites

Metabolites that were significantly regulated among groups were identified using VIP ≥1 and absolute Log_2_FC (│Log_2_FC│≥1). The values of the VIP were obtained based on the results of OPLS-DA, which was produced with the R package MetaboAnalyst R and also provided the score plots and permutation plots. Prior to OPLS-DA, all data were mean-centered and log transformed (log_2_). A permutation test with 200 permutations was conducted to prevent overfitting.

### Analysis of enrichment and KEGG annotation

The KEGG Compound database (http://www.kegg.jp/kegg/compound/) was used to annotate the resulting metabolites, which were subsequently mapped to the KEGG Pathway database (http://www.kegg.jp/kegg/pathway.html). Pathways with significantly regulated metabolites were then inputted into MSEA (metabolite sets enrichment analysis), and the p-values from a hypergeometric test were used to assess their significance.

### RNA extraction and transcriptome sequencing

Total RNA was extracted from soybean samples using the RNAprep Pure Plant Kit (TSINGKE, TSP412). RNA quantification and cDNA library construction were performed according to the methods described by Lu et al. (2018). The libraries were sequenced using Illumina NovaSeq S6000. Three biological replicates were included in each experiment.

### Quality control of data

Fast p v 0.19.3 was used to sort the original data, and filter DNA sequencing reads with adapters. Reads with Ns (unknown base calls) higher than 10% of base read numbers were discarded, as were sequencing reads with >50% low-quality (Q ≤20). The resulting clean reads were used to perform all subsequent analyses.

### Mapping reads to reference genomes

Reference genomes and their related annotation files were obtained from the designated website, while HISAT v2.1.0 was used to generate the index. Clean reads were cross-referenced with the reference genome (Wm82.a2.v1).

### Quantifying levels of gene expression

FeatureCounts v1.6.2 was used to analyze gene alignment, after which gene length was used to establish the FPKM (fragments per kilobase of transcript per million mapped reads) for each gene, which is the most common method of assessing levels of gene expression.

### Analyzing differences

The differential expression between the two groups was analyzed using DESeq2 v1.22.1, while the *P* value was corrected with the Benjamini & Hochberg method. The resulting *P* values and |log_2_ fold-change| were used to categorize significant differences in gene expression. Differentially expressed genes were assessed according to the following parameters: corrected *P*-value of 0.05 and absolute fold change ≥2 (Mao et al., 2005).

### Analysis of differential gene enrichment

The hypergeometric test was used to perform enrichment analysis, while DEG enrichment in KEGG pathways was assessed with KOBAS software (Mortazavi et al., 2008). GO (Gene Ontology) was performed using the GOseq (v3.10.1) (Götz et al., 2008).

## Results

### Metabolome profiling

**Figure 1:**
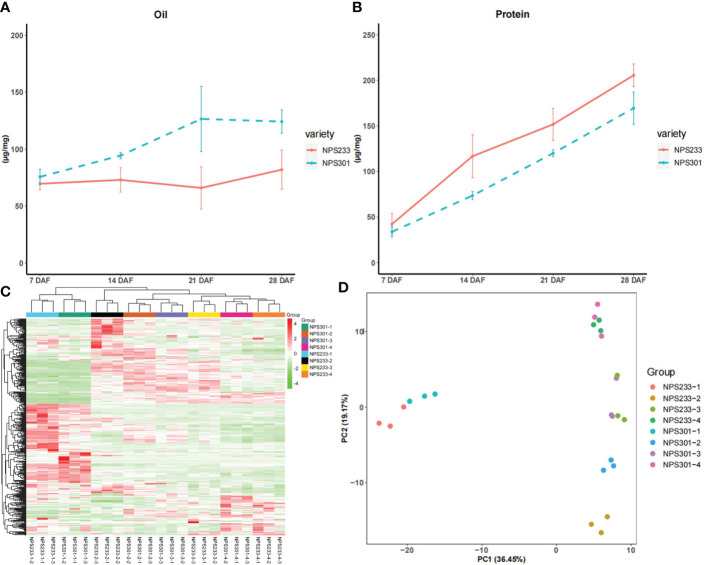
Phenotypic difference and 392 detected metabolites between ‘NSP233’ and ‘NPS301’. **(A)** Protein content of 7 DAF, 14 DAF, 21 DAF and 28DAF. **(B)** Oil content of 7 DAF, 14 DAF, 21 DAF and 28 DAF. **(C)** The hierarchical clustering analysis of the 24 soybean seed samples. **(D)** Principal component analysis of the seed samples, each circle represents a sample. For hierarchical clustering analysis, normalized signal intensities of metabolites (unit variance scaling) are visualized as a color spectrum.7 DAF, NPS233-1 and NPS301-1; 14 DAF, NPS233-2 and NPS301-2; 21 DAF, NPS233-3 and NPS301-3; 28 DAF, NPS233-4 and NPS301-4.

To generate comprehensive metabolic regulatory networks of soybean seed at different developmental periods, we collected soybean seed samples from two soybean varieties at 7 days after flowering (DAF), 14 DAF, 21 DAF, and 28 DAF. Seeds of the soybean variety ‘NPS233’ accumulate more protein and less oil than seeds of ‘NPS301’ at all four seed developmental periods (**Figures 1A, B**). The experiment consisted of three biological replicates, each of which was a pool of seeds from five plants.

The metabolomes of the 24 samples were profiled using the widely-targeted UPLC-MS/MS metabolic profiling approach. We detected 392 compounds that could be grouped into 11 classes, including 25 alkaloids, 58 amino acids and derivatives, 52 flavonoids, 68 lipids, 39 nucleotides and derivatives, 32 organic acids, 30 phenolic acids, 23 terpenoids, 16 saccharides and alcohols, 14 vitamins, and 35 other compounds (**Table S1**). Among the metabolite classes, lipids, amino acids and derivatives, and flavonoids were the most abundant.

We performed hierarchical clustering analysis of the 24 samples (7, 14, 21, and 28 DAF for the two varieties), and the results showed that the three biological replicates from each developmental stage grouped together, which suggested that the generated metabolome data was highly reliable (**Figure 1C**). The metabolites were clustered into three main groups, indicating that there are distinct accumulation levels among the samples taken at four stages of seed development (7, 14, 21, and 28 DAF).

The results of principal component analysis (PCA) grouped the eight samples into four discreet clusters (**Figure 1D**). The first principal component (PC1, 36.45%) effectively separated the 7 DAF samples from the other three samples, implying that there are changes in metabolite accumulation during soybean seed development.

### Analysis of the differentially accumulated metabolites

To identify the differentially accumulated metabolites (DAMs) between each pairwise comparison of soybean seed samples, we analyzed three biological replicates of seeds from the two soybean varieties at four different growth periods (NPS233-1, NPS233-2, NPS233-3, and NPS233-4; NPS301-1, NPS301-2, NPS301-3, and NPS301-4). Metabolites with variable importance in projection (VIP) ≥1 and fold-change ≥2 or ≤0.5 were considered to be DAMs. We performed two separate types of analyses of the DAMs; the first between the different growth periods of the same variety, and the second between the two varieties at the same growth period. As a result, 315 DAMs were detected; in the first group of analyses these included 209, 203, and 203, and 179, 179, and 192 DAMs in NPS233-1 vs. NPS233-2, NPS233-1 vs. NPS233-3, and NPS233-1 vs. NPS233-4, and NPS301-1 vs. NPS301-2, NPS301-1 vs. NPS301-3, and NPS301-1 vs. NPS301-4 (**Table S2**). In the second group of analyses, we detected 69, 104, 45, and 37 DAMs in the NPS301-1 vs. NPS233-1, NPS301-2 vs. NPS233-2, NPS301-3 vs. NPS233-3, and NPS301-4 vs. NPS233-4 comparisons, respectively (**Table S2**). Among the DAMs, we detected 49 flavonoids, 48 lipids, and 44 amino acids and derivatives, and these were the most abundant.

Comparative analysis of the six groups of DAMs identified 274 and 235 DAMs in the NPS233 and NPS301 comparisons within the groups of seed samples, respectively. Moreover, 69 and 30 metabolites were exclusively differentially accumulated in the NPS233 and NPS301 groups, respectively. There were 20 differentially accumulated lipids and six differentially accumulated amino acids and derivatives in the NPS233 groups; we found that one phospho-sugar and one sugar acid, D-fructose 6-phosphate and D-glucuronic acid, were differentially accumulated exclusively in NPS233. Also, one lipid and five amino acids and derivatives were exclusive to the NPS301 groups, but no differentially-accumulated saccharides were found.

Furthermore, we investigated DAMs with different accumulation patterns, which included some intermediates of the TCA cycle; for example, α-ketoglutaric acid, a ketone derivative of glutaric acid, was up-regulated in NPS233 versus NPS301 in developmental period 2 (14 DAF). α-ketoglutarate is its carboxylate and is also known as 2-oxoglutarate; it is a keto acid generated by deaminating glutamate and an intermediate during the Krebs cycle.

We next performed KEGG (Kyoto Encyclopedia of Genes and Genomes) pathway enrichment analysis. The top enriched KEGG terms annotated for all the comparisons were “flavone and flavonol biosynthesis”, “pyrimidine metabolism”, “isoflavonoid biosynthesis”, “biosynthesis of amino acids”, “biosynthesis of secondary metabolites”, “ABC transporters”, “zeatin biosynthesis”, and “aminoacyl-tRNA biosynthesis” (**Figure S1**).

In addition, DAM comparisons between the samples from NPS301 revealed an enrichment of KEGG terms related to “cyanoamino acid metabolism,” “2-oxocarboxylic acid metabolism”, and “pyruvate metabolism”. The top enriched KEGG terms in the comparisons of the NPS233 samples were “flavonoid biosynthesis”, “folate biosynthesis”, “galactose metabolism”, “biotin metabolism”, “alanine, aspartate and glutamate metabolism”, and “cysteine and methionine metabolism” (**Figure S1**).

### Differentially expressed gene analysis

To investigate the molecular mechanisms underlying the regulation of protein and oil biosynthesis in the soybean seed developmental periods, we performed RNA-seq analysis for the two varieties. A total of 47.0 million clean DNA sequencing reads were generated, of which 94.79% had a Phred quality score of Q30 or greater (**Table S3**). The mapping statistics of the sequencing libraries are summarized in **Table S4**. We identified 1,176 novel genes, of which 394 were successfully annotated. In total, 41,036 genes were found to be expressed in at least one sample (**Table S5**).

**Figure 2:**
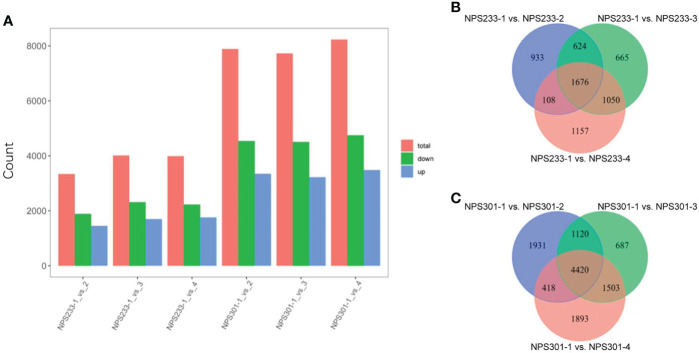
Differentially expressed genes in the six compared groups from NPS233 and NPS301. **(A)** Number of DEGs identified through the comparative analysis. **(B, C)** Venn diagrams depicting the DEGs between pairwise comparisons of seed samples from NPS233 and NPS301.

The dataset was used to identify differentially expressed genes (DEGs). We performed two separate analyses of the DEGs; the first was between the different growth periods of the same variety, and the second was between the two varieties at the same growth period. The pairwise comparisons of samples from NPS301 and NPS233 detected 12,906 DEGs in total (**Table S6**). The pairwise comparisons NPS233-1 vs. NPS233-2, NPS233-1 vs. NPS233-3, and NPS233-1 vs. NPS233-4 identified 3,341 (1,887 upregulated; 1,454 downregulated), 4,015 (2,314 upregulated; 1,701 downregulated) and 3,991 (2,228 upregulated; 1,763 downregulated) DEGs, respectively. The pairwise comparisons NPS301-1 vs. NPS301-2, NPS301-1 vs. NPS301-3, and NPS301-1 vs. NPS301-4 identified 7,889 (4,539 upregulated; 3,350 downregulated), 7,730 (4,505 upregulated; 3,225 downregulated) and 8,234 (4,747 upregulated; 3,487 downregulated) DEGs, respectively (**Figure 2A**; **Table S6**).

We identified 1,676 DEGs across the three compared groups from NPS233 and 4,420 DEGs from the comparative analysis of the NPS301 samples (**Figures 2B, C**), suggesting that these core conserved DEGs may be associated with the accumulation of soybean protein and oil contents, respectively.

We detected 655 DEGs through pairwise comparisons between the two soybean varieties at the same growth period, including 242, 366, 236, and 169 DEGs in NPS301-1 vs. NPS233-1, NPS301-2 vs. NPS233-2, NPS301-3 vs. NPS233-3, and NPS301-4 vs. NPS233-4, respectively (**Table S6**).

To further analyze the potential function of these DEGs, we conducted a KEGG pathway enrichment analysis. The top enriched KEGG terms annotated for all the compared groups were “carbon metabolism”, “glycerolipid metabolism”, “linoleic acid metabolism”, “plant hormone signal transduction”, “biosynthesis of secondary metabolites”, “glycosylphosphatidylinositol (GPI)-anchor biosynthesis”, “beta-alanine metabolism”, and “other glycan degradation”.

Notably, the DEGs in the three NPS233 comparison groups were enriched in the KEGG terms “biosynthesis of amino acids”, “ribosome”, “glycolysis/gluconeogenesis”, “nitrogen metabolism”, “fatty acid degradation”, and “pantothenate and CoA biosynthesis”. KEGG pathway enrichment analysis of DEGs in the pairwise comparisons of NPS301 samples indicated that these genes were involved in several metabolic processes including “fatty acid elongation”, “sphingolipid metabolism”, “fatty acid metabolism”, “pyruvate metabolism”, and “citrate cycle (TCA cycle)” (**Figure S2**).

### DEGs that respond to soybean seed development

To gain further insights into gene expression changes that occurred over the four soybean seed developmental periods, we performed k-means clustering of the DEGs based on their expression patterns and obtained 12 clusters (**Figure S3**; **Table S7**).

The DEGs in clusters 1, 6, 7, and 8 showed similar expression trends in which the expression levels gradually decreased after the second development period (samples NPS301-2 and NPS233-2). DEGs in clusters 3 and 12 showed an increase in expression in the first three periods, after which the expression of these DEGs decreased in the fourth period. Notably, the DEGs in clusters 3 and 12 showed different expression levels in NPS233 and NPS301, indicating that the DEGs in cluster 3 may be mainly related to protein content, while the DEGs in cluster 12 may be mainly related to oil content. The relative expression levels of DEGs in clusters 2, 4, 9, 10, and 11 were down-regulated over the four developmental periods. Among them, the DEGs in cluster 11 showed a rapid down-regulation of expression, and the DEGs in clusters 2 and 4 had opposite expression patterns in NPS233 and NPS301, indicating that these DEGs are strongly associated with oil or protein contents in soybean seeds. Finally, expression levels of DEGs in cluster 5 were rapidly up-regulated, which indicated that these genes are involved in soybean seed development.

**Figure 3:**
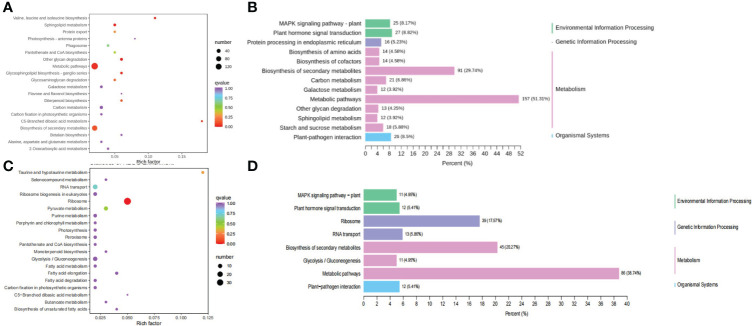
KEGG analysis of the DEGs from cluster 3 and 12. **(A)** KEGG classification of the DEGs from cluster 3. **(B)** KEGG enrichment analysis of the DEGs from cluster 3. **(C)** KEGG classification of the DEGs from cluster 12. **(D)** KEGG enrichment analysis of the DEGs from cluster 12.

These clusters were selected as candidate DEG sets, and four candidate DEG sets containing 2,887 DEGs were further analyzed. We performed a KEGG pathway enrichment analysis of these genes. Enrichment analysis of the DEGs in cluster 3 indicated that they were mainly enriched in protein processing in the endoplasmic reticulum, biosynthesis of the amino acids valine, leucine, and isoleucine, protein export, galactose metabolism, glycosaminoglycan degradation, and other glycan degradation (**Figures 3A, B**), implying that the DEGs in cluster 3 may be mainly related to protein biosynthesis. *Glyma.06G169700* in cluster 3, which was functionally annotated as encoding acetolactate synthase 3, was found to be highly expressed. Acetolactate synthase catalyzes the formation of acetolactate from pyruvate, the first step in the synthesis of the branched-chain amino acids (valine, leucine, and isoleucine). KEGG analysis of DEGs in cluster 12 revealed that these genes were associated with the terms “ribosome”, “ribosome biogenesis in eukaryotes”, “pyruvate metabolism”, “fatty acid elongation”, “biosynthesis of unsaturated fatty acids”, “fatty acid degradation”, “fatty acid metabolism”, “peroxisome”, “glycolysis/gluconeogenesis”, and “taurine and hypotaurine metabolism” (**Figures 3C, D**). *Glyma.05G167700* in cluster 12 is a homolog of *acetolactate synthase small subunit 2*, which encodes the regulatory subunit of acetohydroxy-acid synthase and is involved in the feedback inhibition by branched-chain amino acids. The regulatory subunit is required for full enzymatic activity and contains two repeats of ~180 amino acids, each of which is able to partially activate the catalytic subunit. Leucine inhibits the enzyme reconstituted by the first repeat, though isoleucine and valine do not, and no branched-chain amino acid inhibits enzymes reconstituted by the second repeat (Lee and Duggleby, 2001; Lee and Duggleby, 2002).

In addition, DEGs in cluster 2 were mainly enriched in genes for phenylpropanoid biosynthesis, protein processing in endoplasmic reticulum, glycerolipid metabolism, endocytosis, starch and sucrose metabolism, pentose and glucuronate interconversions or phenylpropanoid biosynthesis, glycerolipid metabolism, linoleic acid metabolism, and valine, leucine and isoleucine degradation. *Glyma.03G205700* in cluster 2 is a homolog of *ANT1* in *Arabidopsis thaliana*, and gene that encodes the protein aromatic and neutral amino acid transporter 1 (ANT1). Aromatic and neutral amino acids are translocated by ANT1, including tryptophan, tyrosine, histidine, phenylalanine, valine, proline, glutamine, leucine, and arginine (Chen et al., 2001). KEGG analysis of DEGs in cluster 4 showed that these genes are involved in pyruvate metabolism, phenylalanine metabolism, phenylpropanoid biosynthesis, pentose and glucuronate interconversions, phenylalanine, tyrosine and tryptophan biosynthesis, tyrosine metabolism, the pentose phosphate pathway, and α-Linolenic acid metabolism (**Figure S4**). *Glyma.04G236900* in cluster 4 was annotated as glutamate synthase [NADH], and this enzyme is involved in glutamate biosynthesis. These results suggest that the DEGs are related to hormone signal transduction, secondary metabolite biosynthesis or phenylpropanoid biosynthesis, amino acid biosynthesis, and glycerolipid metabolism.

### DEGs involved in FA biosynthesis

**Figure 4:**
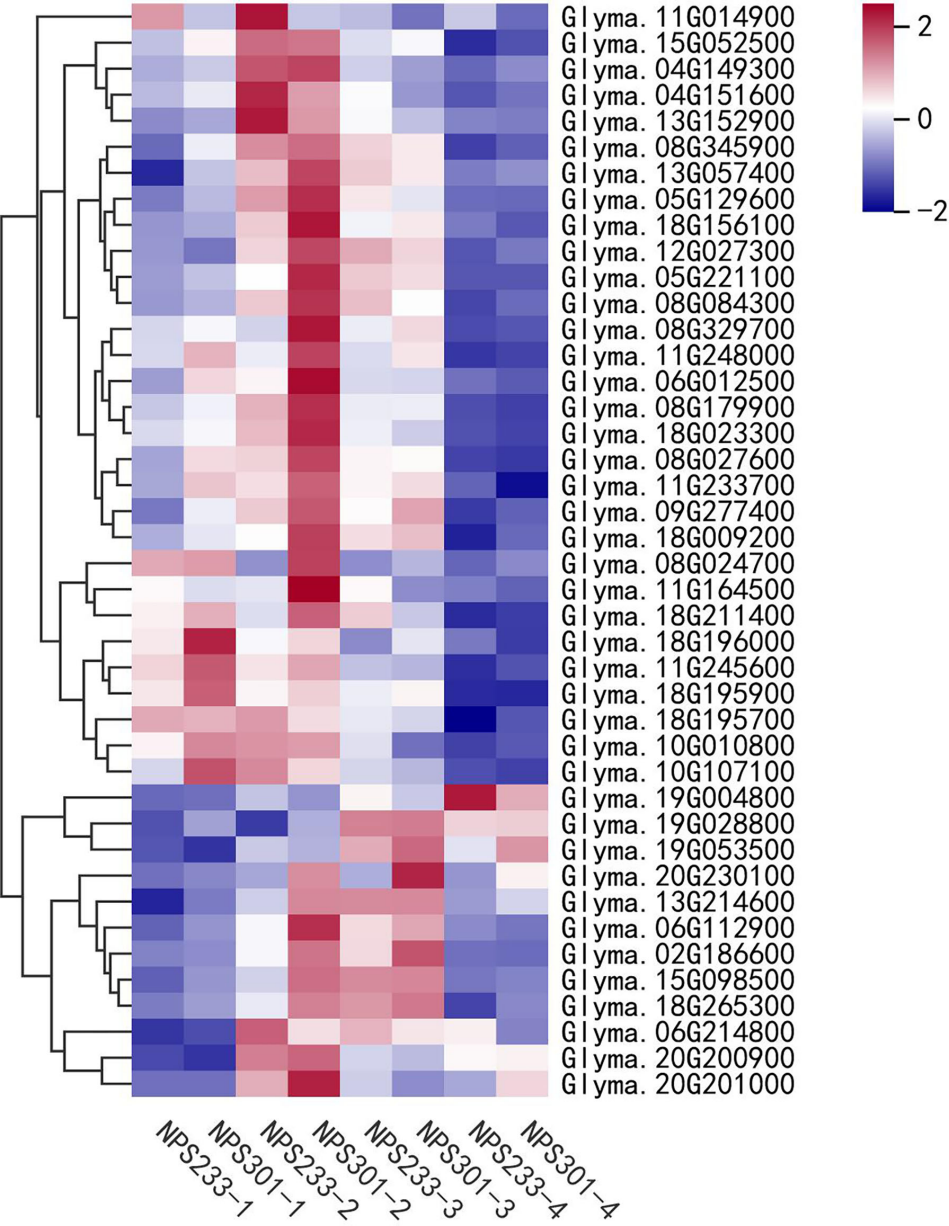
Expression patterns of DEGs involved in lipid biosynthesis and signal transduction pathways in soybean seed development. Heatmap showing the expression patterns of the candidate genes involved in the regulation of oil content accumulation in soybean seed. *Glyma.11G014900*, acyl CoA binding protein; *Glyma.15G052500*, hydroxyacyl-ACP dehydrase; *Glyma.04G149300*, ketoacyl-CoA synthase; *Glyma.04G151600*, fatty acyl thioesterase B; *Glyma.13G152900*, acyl CoA binding protein; *Glyma.08G345900*, enoyl-ACP reductase; *Glyma.13G057400*, heteromeric acetyl CoA carboxylase, biotin carboxyl carrier protein; *Glyma.05G129600*, ketoacyl-ACP synthase I; *Glyma.18G156100*, enoyl-ACP reductase; *Glyma.12G027300*, enoyl-ACP reductase; *Glyma.05G221100*, heteromeric acetyl CoA carboxylase, biotin carboxylase subunit; Glyma.08G084300, ketoacyl-ACP synthase I; *Glyma.08G329700*, acyl-CoA synthase; *Glyma.11G248000*, ketoacyl-ACP reductase; *Glyma.06G012500*, ketoacyl-CoA synthase; *Glyma.08G179900*, hydroxyacyl-ACP dehydrase; *Glyma.18G023300*, biotin/lipoyl attachment domain-containing protein; *Glyma.08G027600*, heteromeric acetyl CoA carboxylase, biotin carboxylase subunit; *Glyma.11G233700*, biotin/lipoyl attachment domain-containing protein; *Glyma.09G277400*, ketoacyl-ACP synthase III; *Glyma.18G009200*, ketoacyl-ACP reductase; *Glyma.08G024700*, ketoacyl-ACP synthase I; *Glyma.11G164500*, malonyl CoA-ACP malonyltransferase; *Glyma.18G211400*, ketoacyl-ACP synthase III; *Glyma.18G196000*, heteromeric acetyl CoA carboxylase, carboxyltransferase alpha subunit; *Glyma.11G245600*, ketoacyl-CoA reductase; *Glyma.18G195900*, heteromeric acetyl CoA carboxylase, carboxyltransferase alpha subunit; *Glyma.18G195700*, heteromeric acetyl CoA carboxylase, carboxyltransferase alpha subunit; *Glyma.10G010800*, ER long-chain acyl- CoA synthetase; *Glyma.10G107100*, glycerol 3 phosphate dehydrogenase; *Glyma.19G004800*, Oleosin; *Glyma.19G028800*, heteromeric acetyl CoA carboxylase, biotin carboxyl carrier protein; *Glyma.19G053500*, glycerol 3 phosphate dehydrogenase; *Glyma.20G230100*, acyl carrier protein; *Glyma.13G214600*, acyl carrier protein; *Glyma.06G112900*, plastidic long-chain acyl- CoA synthetase; *Glyma.02G186600*, glycerol 3 phosphate dehydrogenase; *Glyma.15G098500*, acyl carrier protein; Glyma.18G265300, heteromeric acetyl CoA carboxylase, biotin carboxyl carrier protein; *Glyma.06G214800*, ketoacyl-CoA synthase; *Glyma.20G200900*, Caleosins; *Glyma.20G201000*, Caleosins. The gene per row is Z-score standardized.

To evaluate the expression of FA biosynthetic genes, we investigated the expression patterns of DEGs involved in lipid biosynthesis and signal transduction pathways (**Figure 4**). Most genes related to the *de novo* FA biosynthesis pathway were up-regulated in NPS301 during the first three seed developmental periods (7, 14, and 21 DAF), including the subunits of acetyl-CoA carboxylases and the FA synthase complex. Notably, several genes were down-regulated in NPS301, including one gene for fatty acyl thioesterase B (FATB) (*Glyma.04G151600*), two acyl-CoA binding protein genes (*Glyma.11G014900* and *Glyma.13G152900*), and one oleosin gene (*Glyma.19G004800*). Also, the expression of one ketoacyl-CoA synthase gene (*Glyma.06G214800*) was slightly down-regulated in NPS301. Moreover, a gene encoding ketoacyl-ACP synthase I (*Glyma.08G024700*) was up-regulated >5-fold in NPS301-2 versus NPS233-2, and an acyl carrier protein gene (*Glyma.20G230100*) was highly expressed in the latter three developmental periods (14, 21, and 28 DAF) in NPS301.

### DEGs involved in the Krebs cycle and amino acid biosynthesis

**Figure 5:**
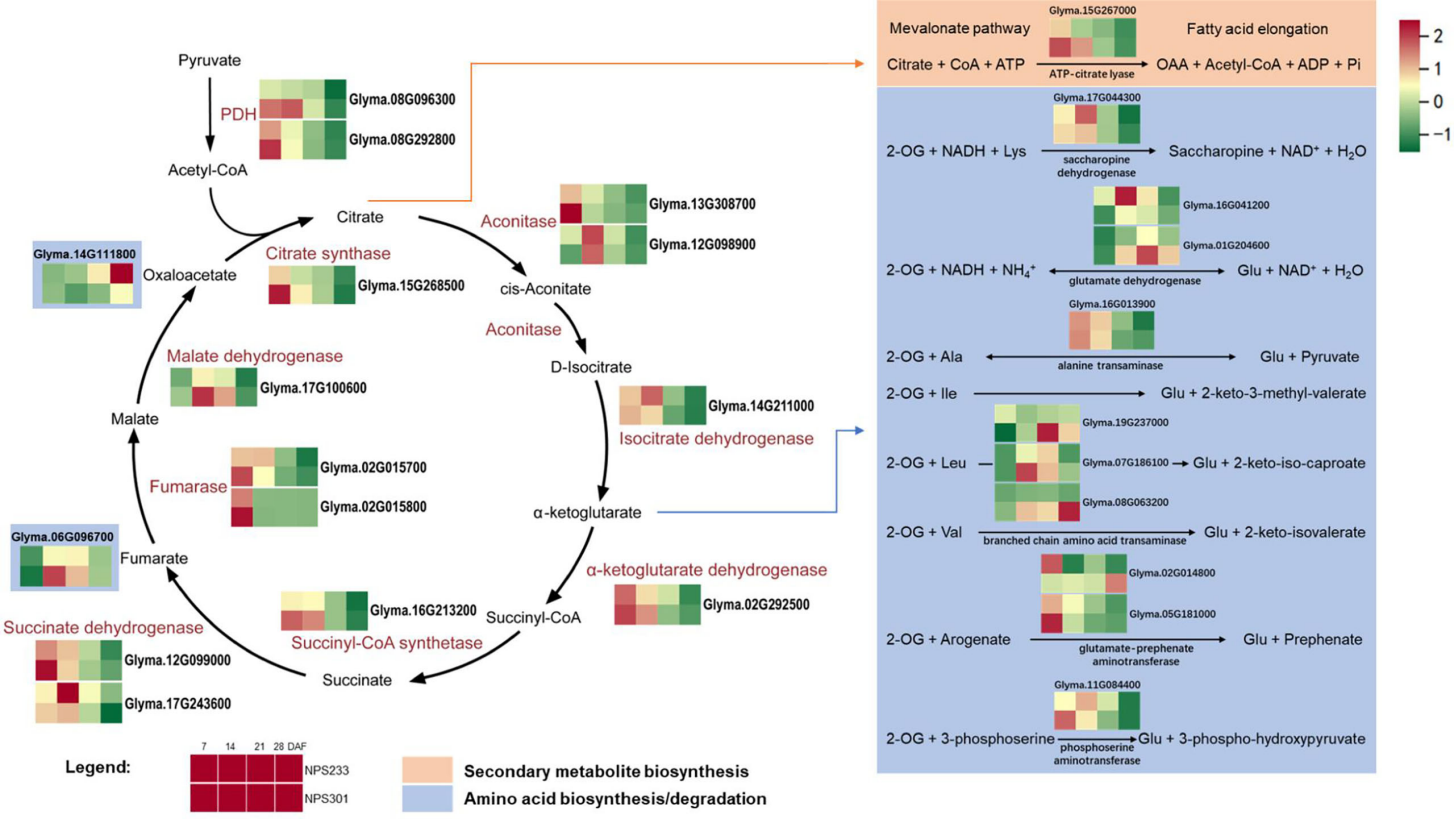
DEGs involved in Krebs cycle and the amino acids metabolic network it embedded in. Reactions are shown that consume or produce TCA cycle intermediates. For simplicity, co-enzymes are omitted from the TCA cycle. For each gene, squares denote expression patterns in each variety (see legend). *Glyma.14G111800*, aspartate aminotransferase, Asp + 2-Oxoglutarate <-> Oxaloacetate + Glu, *Glyma.06G096700*, argininosuccinate lyase, Argininosuccinate -> Arg + Fumarate. The gene per row is Z-score standardized.

To identify putative genes involved in the regulation of protein and oil contents in developing soybean seeds, we analyzed the expression of Krebs cycle genes and the metabolic network within which it is embedded. As a result, most of the genes that encode Krebs cycle enzymes had higher expression levels in NPS301 than in NPS233 (**Figure 5**). With regard to the reactions that consume or produce TCA cycle intermediates, we investigated the expression of genes that encode enzymes involved in amino acid biosynthesis and degradation, secondary metabolite biosynthesis, and fatty acid elongation. Several genes that putatively function in amino acid biosynthesis pathways were up-regulated in NPS233 (**Figure 5**); and example is *Glyma.16G041200*, which encodes glutamate dehydrogenase 1. *Glyma.02G014800* was annotated as a gene encoding bifunctional aspartate aminotransferase and glutamate/aspartate-prephenate aminotransferase (an *AtAAT* homolog). The *AtAAT* gene is required for the transamination of prephenate to arogenate, and is involved in the aromatic amino acids biosynthesis pathway (Torre et al., 2006). This gene was up-regulated in NPS233 compared to NPS301 in the first developmental period, but down-regulated in the fourth period. Another gene, *Glyma.14G111800*, encodes a homolog of the *aspartate aminotransferase P2* gene in *Lupinus angustifolius*. In *Arabidopsis thaliana*, this gene is related to nitrogen metabolism as well as energy and carbon metabolism, and is important for the metabolizing organic acids related to the Krebs cycle and amino acids (Schultz and Coruzzi, 1995). We also identified several genes that were down-regulated in NPS233 versus NPS301, including an argininosuccinate lyase gene (*Glyma.06G096700*), two putative branched-chain-amino-acid aminotransferase 7 genes (*Glyma.07G186100* and *Glyma.08G063200*), a glutamate dehydrogenase 2 gene (*Glyma.01G204600*), and a branched-chain-amino-acid aminotransferase-like protein 2 gene (*Glyma.19G237000*).

## Discussion

Seed development can be classified into three main stages: the first includes embryo growth, cell division, and morphogenesis; the second includes seed maturation and the accumulation of reserves; and the third includes the desiccation of seeds and subsequent dormancy (Weber et al., 2004). In our study, we performed transcriptomics and metabolomics analyses of soybean seeds in four seed developmental periods. These four developmental periods belong to the first two fundamental stages of seed development.

Metabolites and gene regulatory networks for soybean seed development have been studied in previous reports (Collakova et al., 2013; Peng et al., 2021). More recently, comparative metabolome and transcriptome analyses were performed in the developing seeds of grain and vegetable soybeans at R6 stage, 299 DAMs and and 20,546 DEGs were identified between the two varieties (Chen et al., 2022). Functional enrichment analysis revealed that metabolic pathways, including alanine, aspartate and glutamate metabolism, fatty acid degradation, starch and sucrose metabolism, and flavonoid biosynthesis, were up-regulated in vegetable soybean (Chen et al., 2022), which could partly explain the high-quality of soybean.

**Figure 6:**
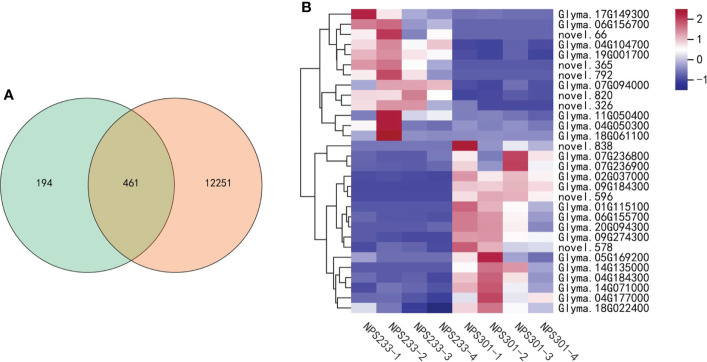
Candidate DEG set belonging to the lipid and amino acids biosynthesis pathways. **(A)** Venn diagram depicting the specific DEGs between the four compared groups. **(B)** Heatmap showing the expression patterns of the candidate genes involved in lipid and amino acids biosynthesis pathways. The gene per row is Z-score standardized.

The purpose of our study is to investigate the mechanisms that regulate soybean seed oil and protein contents accumulation in developing seeds, our results of enrichment analysis were consistent with the previous report. The DEGs were significantly enriched in pathways related to amino acid and fatty acid metabolism, partially explaining the corresponding differentially accumulated metabolites detected between the two varieties.We performed two separate analyses of differentially expressed genes (DEGs); the first was between different growth periods of the same variety, and the second was between the two varieties at the same growth period. The pairwise comparisons of samples from the four different growth periods identified 12,712 DEGs, and the comparisons of groups from different varieties at the same developmental periods identified 655 DEGs. Among them, there are 461 DEGs common to both comparisons, and 194 genes were exclusively differentially expressed in the comparisons between the two soybean varieties (**Figure 6A**). We mapped the selected set of genes to the lipid biosynthesis and amino acids biosynthesis pathways to determine their expression patterns. A total of 30 genes were selected, and this candidate DEG set was then used in further analyses (**Figure 6B**).

Among these 30 genes, two (*Glyma.04G050300* and *Glyma.04G104700*) are involved in leaf senescence delay; *Glyma.04G050300* is annotated as a zinc finger CCCH domain-containing protein 2 (an *OsC3H2* homolog), and *Glyma.04G104700* is annotated as encoding an acyltransferase-like protein (homologous to *At1g54570*). *OsC3H2* may repress the role of jasmonic acid (JA) signaling in promoting leaf senescence and the regulation of panicle development and the pollination/fertilization processes (Kong et al., 2006). Acyltransferase contributes to the synthesis of fatty acid phytyl ester in chloroplasts, which is essential for maintaining the integrity of the photosynthetic membrane during abiotic stress and senescence (Lippold et al., 2012). Expression of both genes was up-regulated in the second seed developmental period in NPS233 versus NPS301, implying that delayed leaf senescence allows more protein to accumulate in the seeds. Previous studies have shown that addition to the *de novo* synthesis of amino acids in seed tissues as they develop an important source of additional free amino acids are those synthesized in vegetative tissues and then transported to seed tissues. The large-scale migration of free amino acids to seeds is related to leaf senescence, where leaves degrade their proteins, producing available free amino acids that can move to develop seeds (Fernie and Hoefgen, 2013; Cohen et al., 2017; Watanabe et al., 2017).

Another gene, *Glyma.17G149300*, is a homolog of *CKB2*. *CKB2* encodes a casein kinase II subunit beta-2, which is involved in the regulation of the basal catalytic activity of the alpha subunit. The tetrameric holoenzyme CK2 has two alpha and two beta subunits and is responsible for phosphorylating the transcription factor PIF1 once it’s exposed to light. This induces proteasome-dependent PIF1 degradation and promotes photomorphogenesis (Bu et al., 2011). *Glyma.17G149300* expression was up-regulated in NPS233 versus NPS301 in the first developmental period, indicating that photomorphogenesis in NPS233 occurs earlier than in NPS301.

In addition, the expression of two seed desiccation-related genes, *Glyma.07G236800* and *Glyma.07G236900*, was up-regulated in the third period in NPS301 versus NPS233. These two genes were annotated as encoding desiccation-related protein pcC13-62, and quantitative analysis demonstrated that there is a lower level of pcC13-62 transcript accumulation in species prone to desiccation than in those that tolerate desiccation (Giarola et al., 2018). Synthesizing seed-storage proteins still happens in the third seed developmental stage (maturation and desiccation), though the oil content typically decreased in this stage (Baud et al., 2002). These results indicate that high-protein soybeans may not tolerate desiccation, and thus accumulate more protein.

In our study, we observed that the gene encoding the desiccation-tolerance protein pcC13-62 was up-regulated in NPS301; at the same time, the gene *CKB2* that promotes photomorphogenesis was highly expressed in NPS233, and two genes involved in the delay of leaf senescence were also up-regulated in NPS233. Moreover, the two soybean varieties that accumulated different protein and oil contents had different maturity dates, with NPS233 maturing earlier. Taken collectively, we propose that the soybean variety NPS233 is more sensitive to desiccation, photomorphogenesis in NPS233 occurs earlier, and leaf senescence is delayed, which could explain why this variety has a higher seed protein content.

There was also a proteasome-related gene identified in the candidate gene set. the 26S proteasome is a protein complex that is responsible for selective, efficient, and processive hydrolysis of intracellular proteins. *Glyma.14G071000*, annotated as encoding the 26S proteasome regulatory subunit 10B homolog A, was up-regulated in NPS301 compared to NPS233. The 26S proteasome is related to the ATP-dependent degradation of ubiquitinated proteins. Few compounds are used to transport and store most nitrogen in plants. The most common compound related to transport in legumes is asparagine (Miflin et al., 1977; Watanabe et al., 1977). An asparagine synthetase gene, *Glyma.18G061100*, was also present in the candidate gene set, and the gene was up-regulated in NPS233 compared to NPS301 in the seed developmental second period, indicating that there is more nitrogen transport in the high protein/low oil soybean variety NPS233.

It is worth noting that in our study, a gene annotated as encoding glutelin type-A 2 (GLUA2), was up-regulated in NPS301 compared to NPS233. *OsGluA2* is involved in the regulation of grain protein content in rice (Yang et al., 2019); it functions as a positive regulator of rice grain protein content and has a pleiotropic effect on rice grain quality. The grain total protein content, as well as the glutelin, albumin, and prolamin contents, is significantly higher in the high grain protein content accession, but globulin is the exception. However, in soybean, globulin is the predominant seed storage protein. The results of our study show that GLUA2 may function differently in soybean compared to rice. The candidate genes used in this study can be manipulated through gene editing or molecular marker-aided selection to improve the quality of soybeans.

One key outcome of our study is a set of potential key candidate genes. Indeed, within the candidate genes are many fatty acid and amino acid metabolism-related genes, including ten acetyl-CoA carboxylase encoding genes, which initiated the *de novo* FA biosynthesis pathway. Metabolites with different pattern of accumulation between the two varieties with respect to amino acid metabolism were identified. For example, α-ketoglutaric acid, was up-regulated in ‘NPS233’ versus ‘NPS301’ in developmental stage 2, whose carboxylate, 2-oxoglutarate provides carbon skeleton for Glu, Gln, proline (Pro) and Arg biosynthesis, and is an intermediate in the Krebs cycle. Some metabolites were not detected, possibly due to the method used in this study, their roles in oil and protein contents accumulation of soybean seed remain to be studied.

## Data availability statement

The original contributions presented in the study are included in the article/**Supplementary Materials**. Further inquiries can be directed to the corresponding author.

## Author contributions

HC designed and supervised this research. WX performed the experiments and obtained transcriptome and metabolome data. QW analyzed the data. QW and WX prepared the draft of the manuscript. WX, WZ, HZ, XL, XYC, QS, YZ and XC revised and improved the manuscript. All authors contributed to the article and approved the submitted version.

## Funding

This work was supported by the National Key Research and Development Program of China (2018YFE0112200), the Key R&D project of Jiangsu Province (BE2019376), the Jiangsu Agriculture Science and Technology Innovation Fund (JASTIF) CX (20)2007, and The Open Competition Project of Seed Industry Revitalization of Jiangsu Province (JBGS [2021]060).

## Conflict of interest

The authors declare that the research was conducted in the absence of any commercial or financial relationships that could be construed as a potential conflict of interest.

## Publisher’s note

All claims expressed in this article are solely those of the authors and do not necessarily represent those of their affiliated organizations, or those of the publisher, the editors and the reviewers. Any product that may be evaluated in this article, or claim that may be made by its manufacturer, is not guaranteed or endorsed by the publisher.

## Supplementary material

Supplementary Figure 1 KEGG enrichment analysis of the differentially accumulated metabolites through the comparative analysis.

Supplementary Figure 2 KEGG enrichment analysis of the differentially expressed genes in NPS233 and NPS301.

Supplementary Figure 3 Dynamics of gene expression during soybean seed development.

Supplementary Figure 4 KEGG classification and enrichment analysis of the differentially expressed genes from the K-means cluster 2 and 4.
