# Supplementary figures and images for: Using transcriptomic and metabolomic data to investigate the molecular mechanisms that determine protein and oil contents during seed development in soybean
Source: Front Plant Sci. 2022 Sep 29;13:1012394. doi: 10.3389/fpls.2022.1012394 (PMC9557928; doi:10.3389/fpls.2022.1012394)

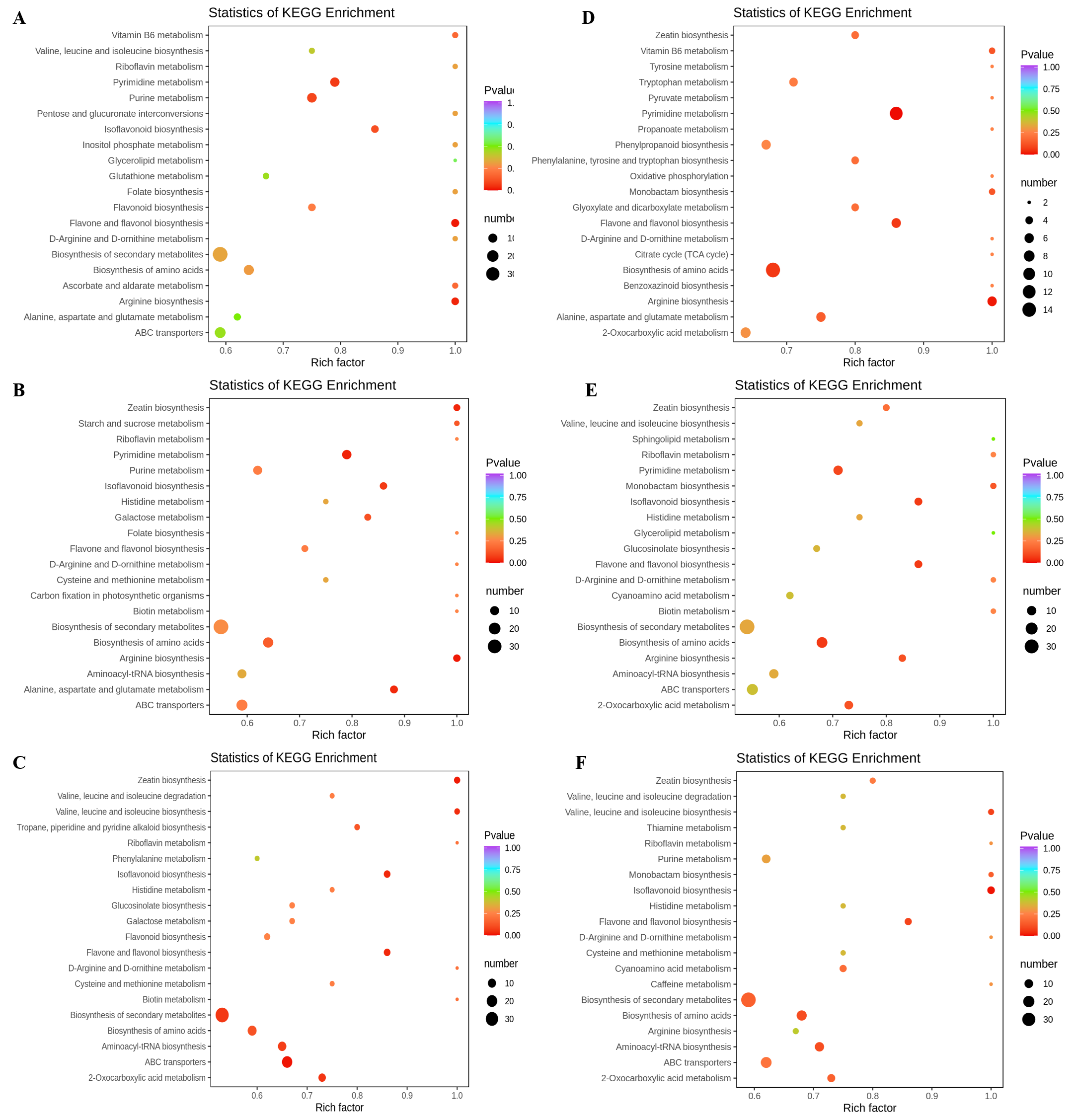

Supplement: Supplementary Figure 1 — KEGG enrichment analysis of the differentially accumulated metabolites through the comparative analysis. [file Image_1.png]

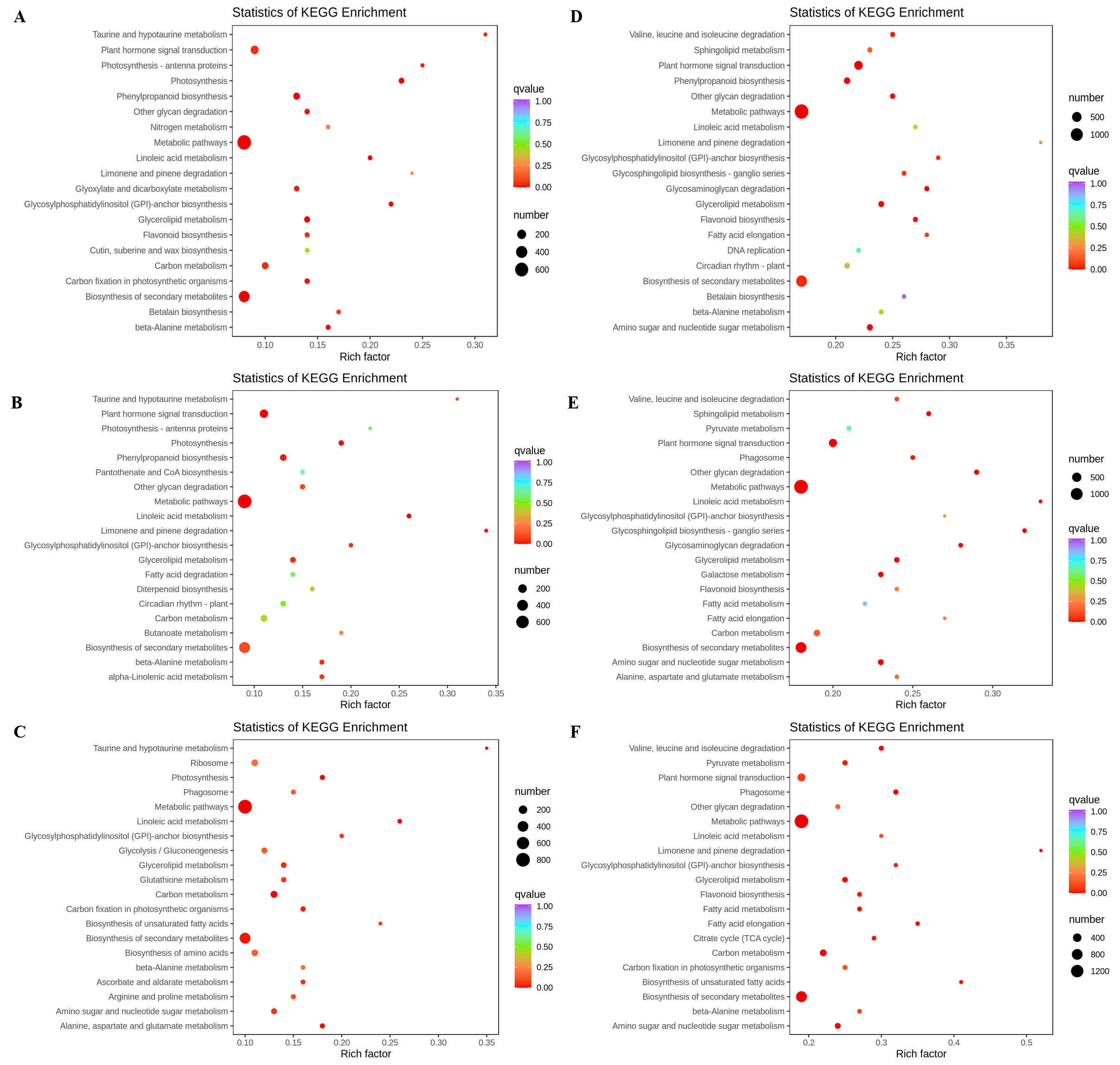

Supplement: Supplementary Figure 2 — KEGG enrichment analysis of the differentially expressed genes in NPS233 and NPS301. [file Image_2.png]

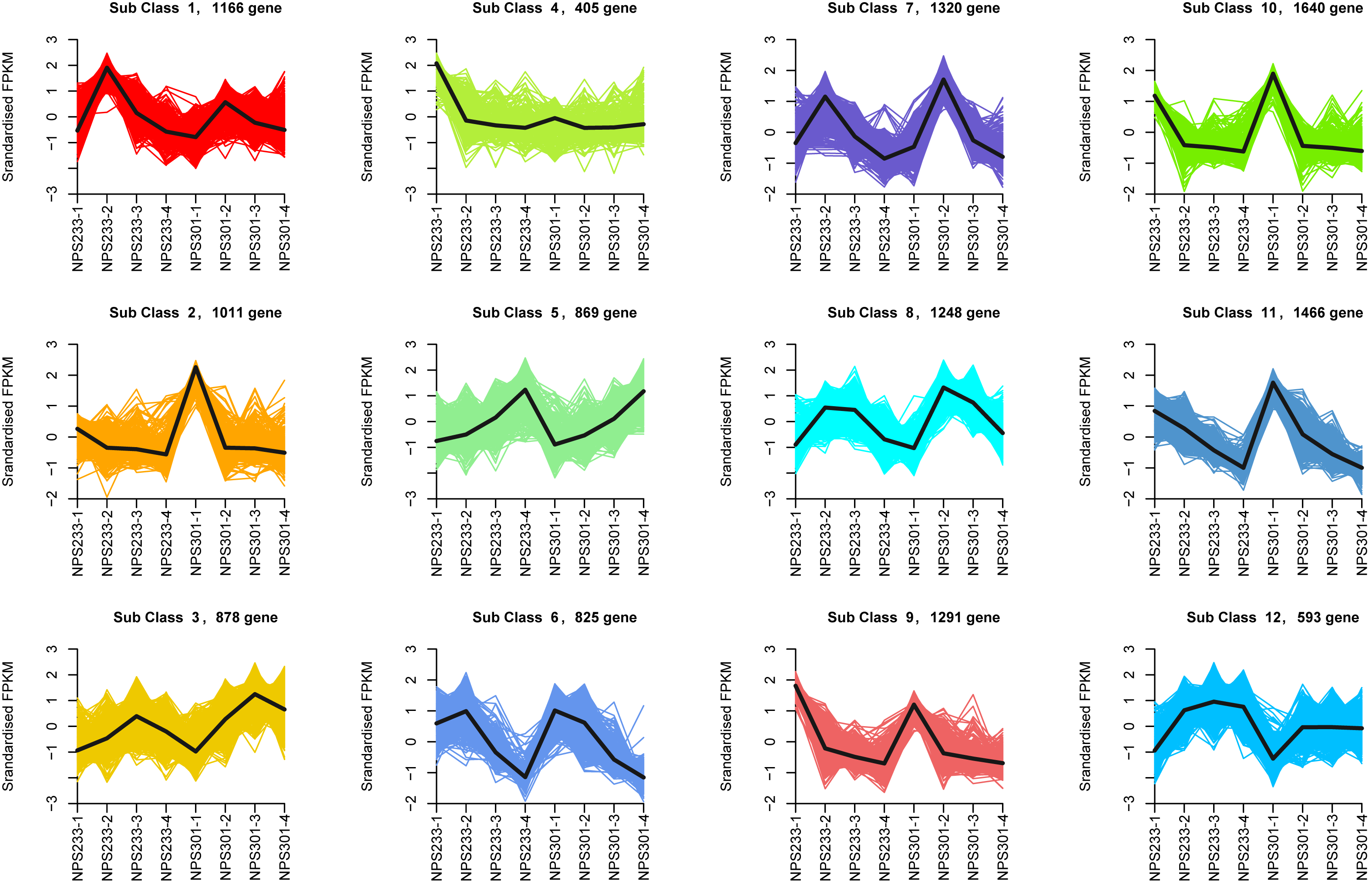

Supplement: Supplementary Figure 3 — Dynamics of gene expression during soybean seed development. [file Image_3.png]

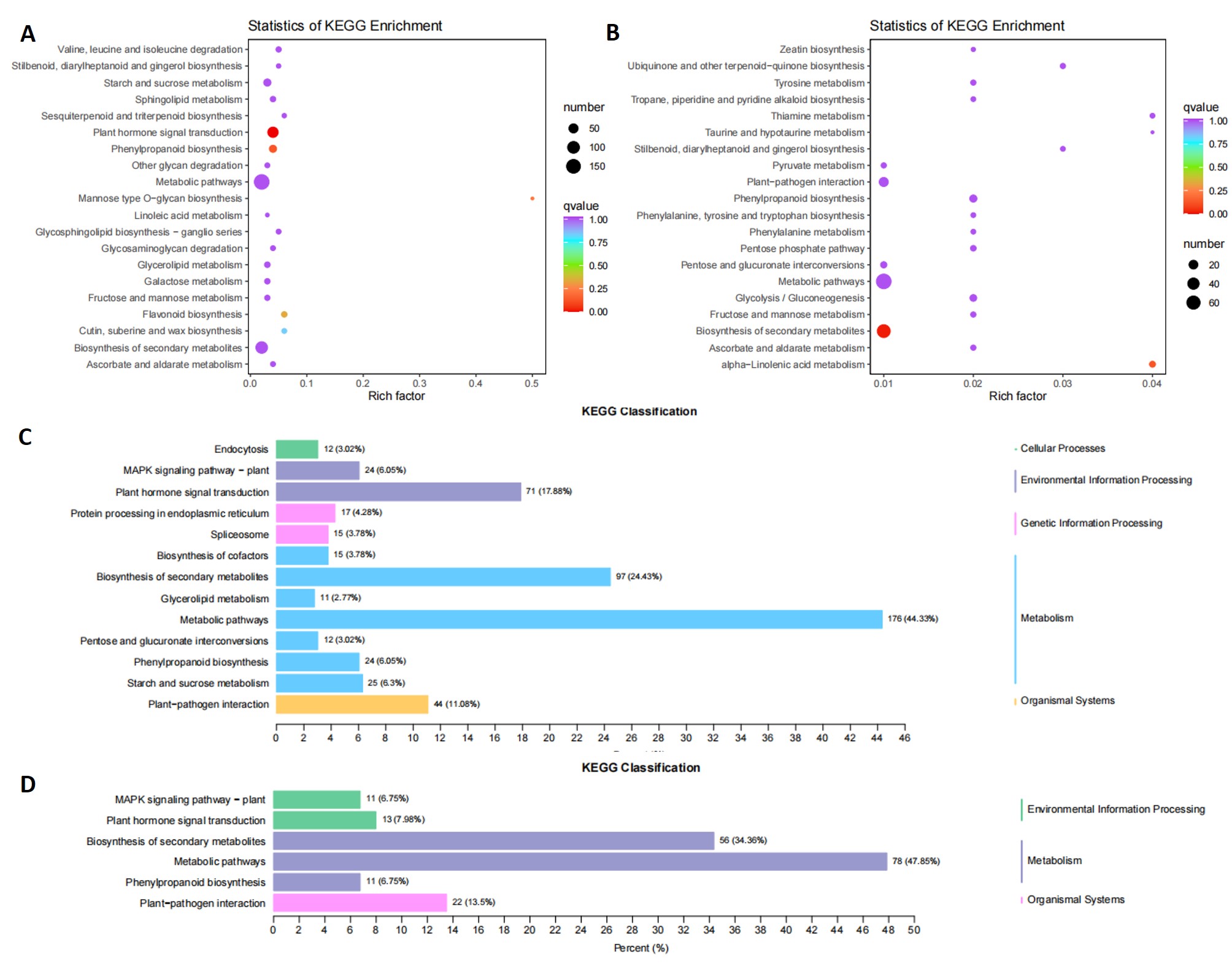

Supplement: Supplementary Figure 4 — KEGG classification and enrichment analysis of the differentially expressed genes from the K-means cluster 2 and 4. [file Image_4.jpeg]
